# Drug Repurposing for COVID-19 by Constructing a Comorbidity Network with Central Nervous System Disorders

**DOI:** 10.3390/ijms25168917

**Published:** 2024-08-16

**Authors:** Jing Qian, Bin Yang, Shuo Wang, Su Yuan, Wenjing Zhu, Ziyun Zhou, Yujuan Zhang, Guang Hu

**Affiliations:** 1 MOE Key Laboratory of Geriatric Diseases and Immunology, Suzhou Key Laboratory of Pathogen Bioscience and Anti-Infective Medicine, Department of Bioinformatics, Center for Systems Biology, School of Life Sciences, Suzhou Medical College of Soochow University, Suzhou 215213, China; qianjing03223@163.com (J.Q.); 2130403023@stu.suda.edu.cn (S.W.);; 2 Experimental Center of Suzhou Medical College of Soochow University, Suzhou 215123, China; 3 Jiangsu Province Engineering Research Center of Precision Diagnostics and Therapeutics Development, Soochow University, Suzhou 215123, China; 4 Key Laboratory of Alkene-Carbon Fibres-Based Technology & Application for Detection of Major Infectious Diseases, Soochow University, Suzhou 215123, China; 5 Jiangsu Key Laboratory of Infection and Immunity, Soochow University, Suzhou 215123, China

**Keywords:** COVID-19, network biology, target module, connectivity map, perturbation response scanning

## Abstract

In the post-COVID-19 era, treatment options for potential SARS-CoV-2 outbreaks remain limited. An increased incidence of central nervous system (CNS) disorders has been observed in long-term COVID-19 patients. Understanding the shared molecular mechanisms between these conditions may provide new insights for developing effective therapies. This study developed an integrative drug-repurposing framework for COVID-19, leveraging comorbidity data with CNS disorders, network-based modular analysis, and dynamic perturbation analysis to identify potential drug targets and candidates against SARS-CoV-2. We constructed a comorbidity network based on the literature and data collection, including COVID-19-related proteins and genes associated with Alzheimer’s disease, Parkinson’s disease, multiple sclerosis, and autism spectrum disorder. Functional module detection and annotation identified a module primarily involved in protein synthesis as a key target module, utilizing connectivity map drug perturbation data. Through the construction of a weighted drug–target network and dynamic network-based drug-repurposing analysis, ubiquitin–carboxy-terminal hydrolase L1 emerged as a potential drug target. Molecular dynamics simulations suggested pregnenolone and BRD-K87426499 as two drug candidates for COVID-19. This study introduces a dynamic-perturbation-network-based drug-repurposing approach to identify COVID-19 drug targets and candidates by incorporating the comorbidity conditions of CNS disorders.

## 1. Introduction

The COVID-19 pandemic, caused by the SARS-CoV-2 virus, has resulted in millions of deaths and continues to pose a significant global public health challenge [1]. The novelty of SARS-CoV-2 and the initial lack of effective drugs and vaccines have led to the development of a wide variety of strategies employed to combat this worldwide pandemic [2]. The treatment of COVID-19 has developed substantially in recent years, significantly reducing mortality rates through improved drug treatments and vaccination [3]. However, the post-acute sequelae of SARS-CoV-2 infection—referred to as long COVID—include several long-term neurologic disorders [4,5]. Even young people with a mild initial illness may experience acute COVID-19 and persistent neuropsychiatric symptoms. Although evidence mainly suggests immune dysfunction, including non-specific neuroinflammation and autoimmune disorders, the pathophysiological mechanisms remain unclear [5]. Therefore, understanding the comorbidities of severe illness and long COVID, and identifying potential therapeutics for long COVID, is an emerging and urgent area in the post-COVID-19 era.

Understanding the molecular bases of disease co-occurrences can improve comorbidity treatment by shedding light on shared pathways and genes [6,7]. Multiple comorbidities [8,9,10,11,12,13], including cancer, atherosclerosis, type 2 diabetes, cardiovascular diseases, and chronic kidney diseases, are associated with the severity and increased mortality of COVID-19, offering new opportunities for drug repositioning for COVID-19 [14]. In particular, central nervous system (CNS) symptoms are common during and after SARS-CoV-2 infection and can persist for a long time [15,16]. Moreover, current evidence supports that SARS-CoV-2 has the capacity to target and invade the CNS [17]. As the COVID-19 pandemic continues, deciphering the comorbidity mechanisms between COVID-19 and CNS disorders has become particularly important [18,19]. Because viral infections are correlated with various CNS disorders, such as Alzheimer’s disease (AD), Parkinson’s disease (PD), multiple sclerosis (MS), and amyotrophic lateral sclerosis (ALS), studying the comorbidity between COVID-19 and CNS disorders is crucial [20,21,22]. This research not only helps to comprehend the enduring effects of COVID-19 but also may uncover new treatment strategies for other viruses [23].

Network-based drug repurposing has emerged as a highly active field of research, further propelled by the urgency of the COVID-19 crisis [24]. A series of useful drug-repurposing tools has been developed, leveraging data-driven modeling and network pharmacology [14]. In this context, network-based proximity analysis [25,26,27] has proven to be an efficient tool for screening potential new medical indications for various drugs that have already been approved and are in clinical use. Compared with artificial intelligence and network diffusion, an integrated algorithm was also employed to rank drugs for their expected efficacies against SARS-CoV-2 [28]. CoVex, an interactive platform, integrates SARS-CoV-2–host interactome data with drug–target interactions, aiding in the understanding of molecular mechanisms and the prioritization of therapeutic candidates [29]. Despite these advancements, no repurposed drugs have been clinically proven against SARS-CoV-2 because of its complex pathological mechanisms [30,31]. Therefore, a more systematic exploration of the molecular details of COVID-19 is necessary [32].

**Figure 1:**
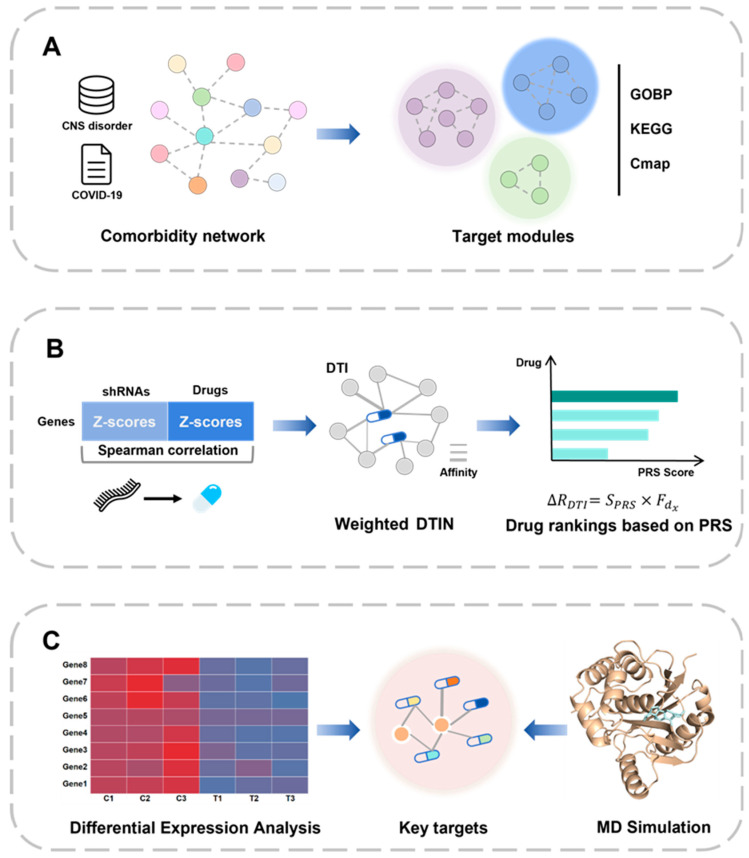
The workflow of this study. (**A**) The construction of a comorbidity network of COVID-19 and CNS disorders and the identification of drug–target modules. (**B**) The construction of weighted drug–target networks based on the target module and the prediction of key targets and ranking drug candidates using a dynamic network-based drug-repurposing method. (**C**) The testing of promising targets and drugs using transcriptome data and molecular dynamics simulations.

In this study, we proposed an integrated system-biology-based method for identifying drug targets and potential drug candidates for patients with long-COVID-related CNS disorders (Figure 1). First, we constructed a comorbidity network by collecting genes and proteins related to COVID-19 and four CNS disorders and identified the drug–target module through functional module detection and annotation and then screened it using connectivity map (CMap) drug perturbation data. Next, we constructed a drug–target network (DTN) based on the target module by combining Spearman correlation calculations between shRNA- and drug-perturbed signature profiles, and the binding affinity was predicted using a deep-learning method. Subsequently, we applied a dynamic network-based drug-repurposing method to the drug–target module to predict potential drug targets and drug candidates. Finally, we tested the identified promising targets and drugs using transcriptome data and molecular dynamics (MD) simulations.

## 2. Results

### 2.1. Construction of the Comorbidity Network

**Figure 2:**
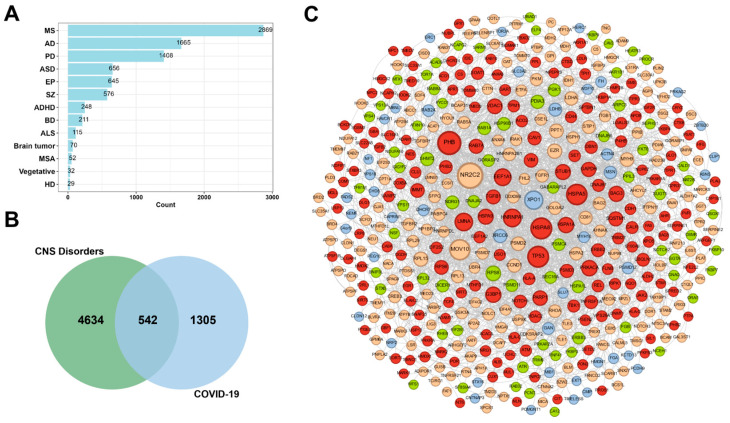
The construction of a comorbidity network based on common genes between COVID-19 and CNS disorders. (**A**) The number of publications on CNS disorders related to COVID-19. (**B**) Venn diagram showing the number of shared genes between COVID-19 and CNS disorders. (**C**) The global topology of the comorbidity network, with orange, green, blue, and red nodes denoting AD, PD, ASD, and multidisease-related genes (see Figure S1 and Table S1), respectively.

To explore the relationships between COVID-19 and CNS disorders, we searched the published literature involving these two different diseases. As shown in Figure 2A, four CNS disorders—MS, AD, PD, and autism spectrum disorder (ASD)—had the highest number of co-reported papers with COVID-19, with counts of 2869, 1665, 1408, and 656, respectively. Thus, these four CNS disorders were selected as the studied comorbidities of COVID-19. We retrieved 3442 genes related to AD, 2415 genes related to PD, 1163 genes related to ASD, and 845 genes related to MS from multiple databases, ultimately obtaining 5176 merging genes associated with these four CNS disorders (Figure S1). In addition, we acquired 1847 high-confidence COVID-19-related proteins from three studies [33,34,35]. By intersecting the CNS disorder gene sets with the COVID-19-related proteins, we identified a total of 542 shared genes (Figure 2B and Table S1). Then, a protein–protein interaction (PPI) network comprising 542 nodes and 4849 edges was constructed based on the shared-comorbidity gene set (Figure 2C).

**Figure 3:**
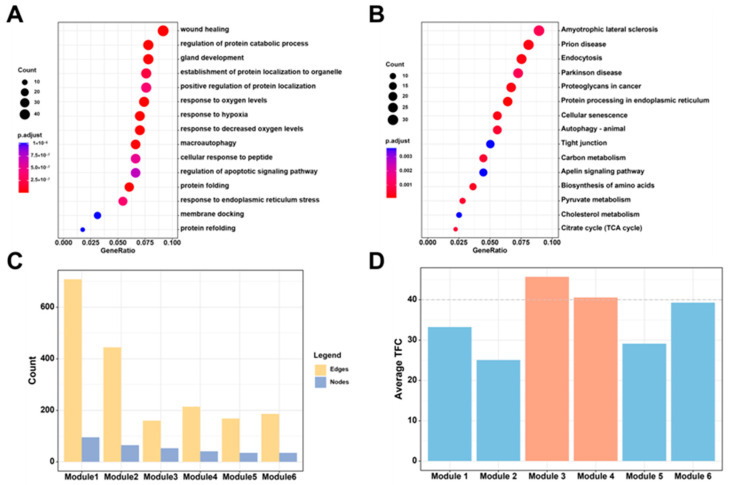
Functional and topological analyses of the comorbidity network. (**A**) Top 15 enriched biological processes associated with the comorbidity network. (**B**) Top 15 enriched KEGG pathways associated with the comorbidity network. (**C**) The node and edge numbers in each module. (**D**) The average TFC score of each module in the comorbidity network.

Further, the functional and topological analyses of the comorbidity network were performed. In the gene ontology biological process (GOBP) enrichment results (Figure 3A and Table S2), the biological process of protein folding was highlighted, reflecting abnormal protein homeostasis observed in CNS disorders. Abnormal protein folding can lead to pathological protein aggregation, which is characteristic of these disorders. Additionally, responses to oxygen levels were implicated in changes in energy metabolism and mitochondrial dysfunction associated with CNS disorders. The Kyoto Encyclopedia of Genes and Genomes (KEGG) pathway enrichment results (Figure 3B and Table S3) indicated associations with pyruvate metabolism and the TCA cycle, both crucial for energy metabolism alterations seen in CNS disorders. Endocytosis, another enriched pathway, plays a critical role in neurotransmitter release and synaptic dysfunction in these disorders. Notably, ACE2-mediated endocytosis facilitates SARS-CoV-2 entry into host cells [36]. Furthermore, the autophagy process, vital for viral particle degradation, is impaired by highly pathogenic of COVID-19 [37]. Accordingly, functional enrichment analysis revealed that these genes were significantly enriched in pathways related to the cell division, cell cycle, DNA replication, angiogenesis, cell migration, and cell differentiation, all of which are well-known hallmarks of both COVID-19 and CNS disorders [38]. However, the biological functions of the comorbidity network are quite broad, with dysregulation of processes, such as pyruvate metabolism and the TCA cycle, also being linked to cancer [39,40]. Therefore, it is necessary to further identify and analyze network modules with more specific biological functions.

Using the ne-PCA method, the comorbidity network was segmented into functional units forming six large modules (Figure S2), named modules 1 to 6 based on the number of nodes (Figure 3C). The node counts for these modules were 95, 65, 53, 41, 34, and 35, respectively. To assess the efficiency of the signal transmission within the module’s PPI network, the average TFC score was calculated (Figure 3D). Modules 3 and 4 exhibited the highest average TFC scores among the six modules. Despite not having the highest number of edges, modules 3 and 4 showed dense edge functions, indicating efficient signal transmission within these modules.

### 2.2. Identification of the Drug–Target Module

**Figure 4:**
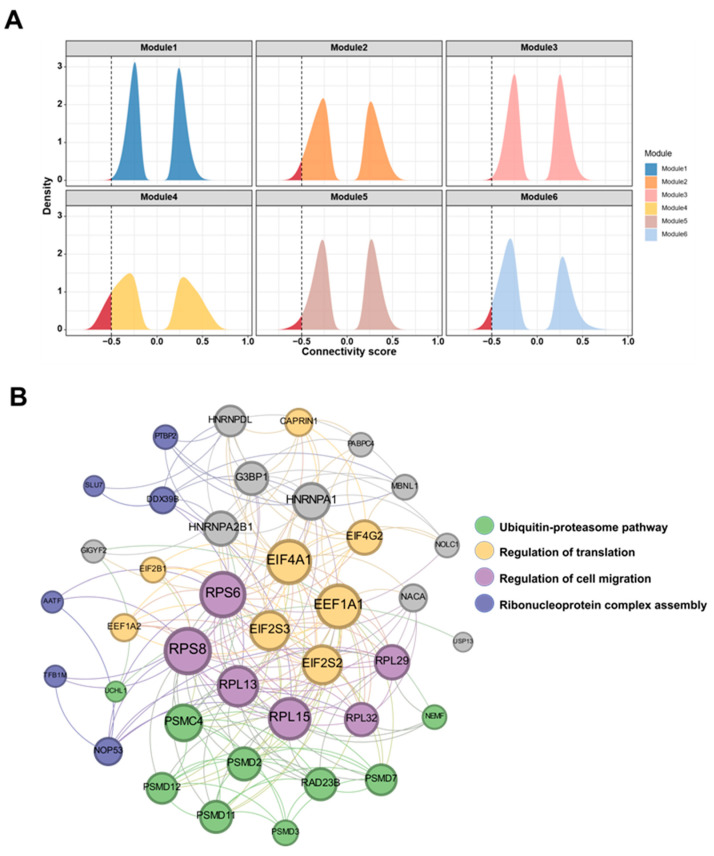
Module analysis. (**A**) Distribution maps of connectivity scores for six modules. The proximity of the drug signature connectivity score to −1 indicates better reversal effects on the expression patterns of the module’s genes (the red area represents connectivity scores of <−0.5). (**B**) Module 4 and its functional annotations, while nodes enriched with different pathways and GO terms are marked by different colors.

By analyzing the connectivity score distribution of all the drugs in the CMap across different modules, it was observed that modules with scores concentrated closer to −1 exhibit higher potential drug responses. The distribution of the drug signature scores for all the modules generally showed a symmetric bimodal shape (Figure 4A), focusing particularly on the negative connectivity score range. Across all the modules, the peak connectivity score was consistently near −0.25. Thus, we used the proportion of drug signatures with connectivity scores distributed between −0.5 and −1 as an indicator to assess the potential drug responses of each module. Module 4 stood out, with the highest proportion in this range, indicating that compounds associated with this module effectively reverse gene expression. This underscores the robust potential of module 4 for targeted therapy applications compared to the other modules.

Module 4 contained 41 genes and 214 PPIs (Figure 4B). GOBP and KEGG pathway enrichment analyses were performed to explore its potential biological functions. According to GO terminology, module 4 was primarily associated with protein synthesis, including cytoplasmic translation, translation initiation, ribosomal protein complex biosynthesis, ribosomal protein complex subunit tissue synthesis, ribosomal protein complex assembly, as well as protein breakdown processes, including proteasome-mediated ubiquitin-dependent protein breakdown metabolism and the regulation of protein breakdown metabolism processes. The key biological pathways enriched in module 4 included the proteasome, Parkinson’s disease, pathways of neurodegeneration, and others. In particular, the ubiquitin–proteasome system serves as the primary pathway for intracellular protein degradation, participating in the degradation of over 80% of the intracellular proteins, promoting DNA damage, the activation of pro-inflammatory pathways, and cellular aging [41].

In summary, the comorbidity network based on shared genes between COVID-19 and CNS disorders was constructed, and the topological and functional analyses provided crucial insights into the complex interactions between COVID-19 and CNS disorders. This underlines the significance of module 4 in shaping therapeutic development strategies aimed at combating the COVID-19 pandemic.

### 2.3. Drug Repositioning for the Target Module

**Figure 5:**
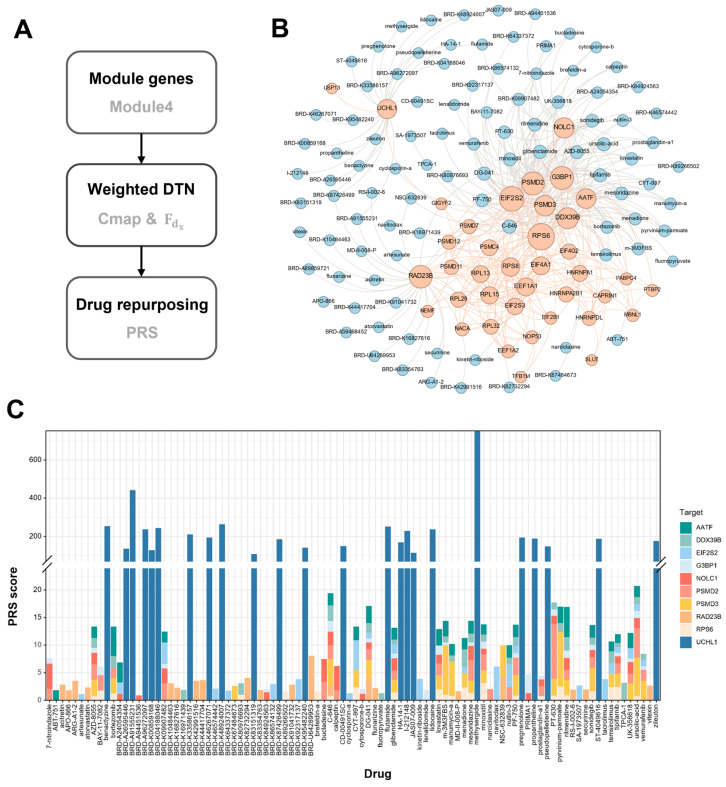
Drug repositioning for module 4. (**A**) The flowchart for the weighted-DTN construction. (**B**) The DTN based on module 4, in which blue and orange nodes represent compounds and target genes, and orange and gray edges represent PPI and compound–target interactions. (**C**) The stacked plot of the PRS scores for the corresponding compounds targeting the ten genes identified in module 4.

Drug repositioning based on target modules primarily involved two steps (Figure 5A). First, CMap was used to construct the non-weighted DTN through the Spearman correlation analysis between the shRNA and drug perturbation feature spectrum, and DeepPurpose was used to calculate the drug–target affinities as weights to construct the weighted DTN. Next, the Gaussian network modeling (GNM) of the weighted DTN was performed and then the perturbation response analysis (PRS) was performed for the elastic network to prioritize drugs and predict crucial drug–target interactions (DTIs) [42].

In module 4, we identified a total of 10 genes (AATF, DDX39B, EIF2S2, G3BP1, NOLC1, PSMD2, PSMD3, RAD23B, RPS6, and UCHL1) with shRNA perturbation expression profiles available from CMap 2020 in lung-related cell lines. We calculated the correlations between the shRNA perturbation profiles of these genes and the compound perturbation profiles, focusing on compounds with correlations in the top 1%. These compounds are presumed to target the proteins encoded by these genes. As a result, we identified 24 compounds targeting each gene. After removing duplicates where the same drug targeted multiple genes, a total of 102 unique compounds were obtained. All these compounds are considered as potential drugs. Consequently, a DTN was constructed comprising 39 protein nodes (of which 11 were targets), 102 drug nodes, 264 DTIs, and 290 PPIs (Figure 5B). Then, we calculated the binding affinity of each DTI to establish the final weighted DTN.

Subsequently, we conducted PRS analysis on the weighted DTN. This method utilizes the GNM to compute the perturbation fraction of individual drugs acting on a target protein within the entire DTN and ultimately aggregates the perturbation fractions of each drug across the different target proteins. In the context of perturbation scores for drug–target protein interaction pairs, a higher score indicates a greater impact of the drug on the DTN of the target protein, which, in turn, signifies the higher importance of the target protein in the network. Our findings revealed that many drugs with high perturbation scores within the DTN based on module 4 targeted UCHL1. Among the top 50 drugs, 24 target ubiquitin–carboxy-terminal hydrolase L1 (UCHL1), significantly outnumbering the other nodes (Figure 5C). Therefore, we speculate that UCHL1 is a key target protein for module 4 and that drugs exert a significant influence on the entire DTN by perturbing UCHL1.

### 2.4. UCHL1 Served as a Potential Drug Target

**Figure 6:**
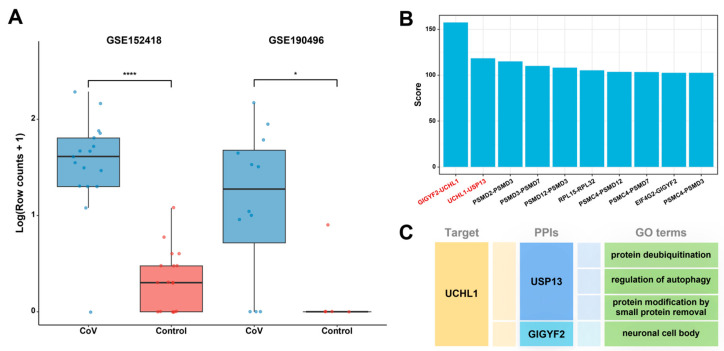
The molecular mechanism of UCHL1 as a potential drug target. (**A**) The expression of the UCHL1 gene was significantly upregulated in the cohort of COVID-19 patients compared to the healthy control group (log2(FC) > 1, *p*-value < 0.05). * *p*-value < 0.05, **** *p*-value < 0.0001. (**B**) Top ten PPIs with the highest TFC scores in module 4, including UCHL1-GIGYF2 and UCHL1-USP13. (**C**) GO terms associated with UCHL1-GIGYF2 and UCHL1-USP13.

By analyzing the publicly available transcriptome datasets, GSE152418 and GSE190496, from COVID-19 patients and healthy controls [43], we observed the significant differential expression patterns of the UCHL1 gene in both datasets (Figure 6A and Figure S3). In the GSE152418 dataset, the expression level of UCHL1 in peripheral blood mononuclear cells (PBMCs) was significantly upregulated in COVID-19 patients (log_2_(FC) = 4.79, *p*-value = 7.2 × 10^−6^), consisting of 12 severe cases, 4 mild cases, and 1 convalescent case. Similarly, in the GSE190496 dataset, UCHL1 expression also showed a significant upregulation trend (log_2_(FC) = 4.67, *p*-value = 0.025) in lung tissue cell samples of COVID-19 patients (11 postmortem cases and 2 survivors). This finding reveals a notable increase in UCHL1 gene expression in COVID-19 patients, providing strong evidence for UCHL1 as a potential drug target.

To further evaluate the potential of UCHL1 as a drug target, we analyzed the PPIs in module 4. The results showed that the TFC scores of GIGYF2-UCHL1 and USP13-UCHL1, two interacting pairs involving UCHL1, ranked first and second in module 4 (Figure 6B). UCHL1 and USP13 jointly participated in the protein deubiquitination process and autophagy regulation, while UCHL1 and GIGYF2 jointly participated in neuronal cell production (Figure 6C). This result emphasizes the importance of protein modification pathways and neuronal cell body generation and further confirms the significant potential of UCHL1 in drug development.

It is worth noting that UCHL1 has been reported in all four CNS disorders. There is a negative correlation between the S18Y variant of the UCHL1 gene and susceptibility to PD, and UCHL1 can promote the neurotoxicity of alpha-synuclein, exacerbating disease progression [44,45]. In AD, oxidative damage to UCHL1 leads to its dysfunction, which, in turn, affects the ubiquitin–proteasome system (UPS), causing dysfunction of the protein clearance network and resulting in neuronal degeneration and synaptic damage [46]. In ASD, mutations in the UCHL1 gene may lead to UPS dysfunction, affecting neural development. The loss or mutation of its function may lead to abnormalities in brain structure and function, including symptoms of ASD, such as deficits in learning ability and social behavior [47]. Research has found that in patients with MS (especially relapse–release-type MS, RRMS), the concentration of UCHL1 in the plasma is significantly higher than that in the healthy control group, indicating that UCHL1 may serve as a sensitive biomarker for the diagnosis of RRMS [48]. In addition, in RRMS patients, the UCHL1 concentration is positively correlated with plasma fibronectin levels. Fibronectin can activate microglia, which respond by producing reactive oxygen species (ROSs), leading to neuronal damage. These findings not only emphasize the importance of UCHL1 in CNS disorders but also help us to understand its mechanism of action as a potential drug target.

### 2.5. Identification of Potential Drug Candidates Based on the UCHL1 Drug–Target Network

In this study, we constructed a drug–target network that unveiled the interactions between UCHL1 and 24 drugs, as illustrated in Figure S4. Among these drugs, nine are medications that have received FDA approval, while another 15 are currently undergoing clinical trials. To delve deeper into the binding patterns and affinities between UCHL1 and these drugs, we performed molecular docking analyses and ranked the drugs based on their predicted docking energies (Table 1). Meanwhile, research into the mechanisms of action (MOAs) of these drugs led us to focus on four, in particular, that are associated with dopamine, namely, pregnenolone, BRD-K62493605, BRD-K87426499, and BRD-A96272097. The predicted docking energies for their interactions with UCHL1 were −7.7, −8.4, −8.1, and −5.2 (kcal/mol), respectively, indicating from moderate to strong interactions. Because of the roles of dopamine in COVID-19 and CNS diseases, we chose these four drug–UCHL1-binding systems for further MD simulations to clearly elucidate the possible interaction mechanisms between potential targets and drugs.

**Figure 7:**
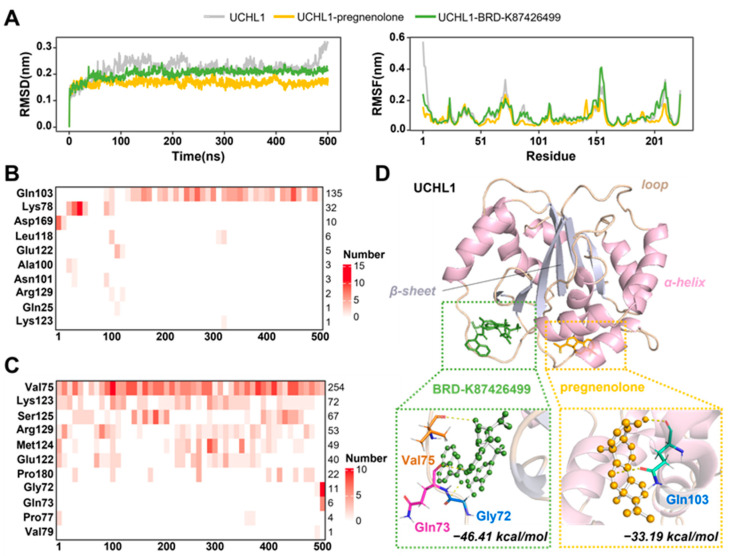
The 500 ns MD simulations of the UCHL1–drug complexes. (**A**) RMSDs and RMSFs of the UCHL1 apo system and the systems of UCHL1 complexed with pregnenolone and BRD-K87426499, respectively. (**B**,**C**) depict the hydrogen-bonding connection between pregnenolone and BRD-K87426499 to UCHL1. Changes in color intensity indicate the frequency and intensity of these interactions. (**D**) The specific conformations of ligand molecules in protein-binding pockets. Important interacting residues and binding free-energies after stabilization are marked.

All-atom 500 ns MD simulations revealed that the complexes formed by pregnenolone and BRD-K87426499 with UCHL1 were more stable, with binding free-energies ($∆G_{MMGBSA}$) of −33.19 and −46.41 kcal/mol (Table S4), respectively. After reaching equilibrium, all four drug-bound systems exhibited lower carbon skeleton shifts compared to the apo system, as analyzed using RMSD (Figure 7A and Figure S5A). Among them, the system bound to pregnenolone demonstrated the smallest carbon skeleton deviations, at approximately 0.17 nm, indicating a higher level of stability. Meanwhile, we observed that the fluctuations in UCHL1 residues were generally minimal in the pregnenolone-bound system, and regions Gly24-Val29, Glu68-Glu 74, and Leu118-Lys135 of UCHL1 demonstrated good stability in both the pregnenolone- and BRD-K87426499-binding systems compared with the others (Figure 7A and Figure S5A). This suggested that these specific regions might play a crucial role in maintaining the structural integrity and stability of the UCHL1–drug complexes. Additionally, in the simulation processes of systems other than BRD-K62493605-UCHL1, stable hydrogen bonds were consistently present (Figure 7B–D and Figure S5B–D). Specifically, a stable hydrogen bond was formed in Gln103 after the stable binding of pregnenolone (Figure 7B,D). In the BRD-K87426499-binding system, Val75 remained closely connected to the drug throughout the entire process, eventually leading to the formation of new hydrogen bonds between Gly72 and Gln 73 (Figure 7C,D). Accordingly, the drug–target network analysis, in conjunction with MD simulations, suggests that two drugs, pregnenolone and BRD-K87426499, act as potential therapeutic agents for COVID-19.

## 3. Discussion

Since the pandemic began, global research has focused on finding drugs to treat COVID-19, primarily aiming to reduce hospitalization and death rates, with less emphasis on improving neurological complications and long COVID. In this study, we employed an integrated computational approach, including data integration, network module and perturbation analyses, gene expression analysis, structural modeling, and MD simulations, to identify and test novel SARS-CoV-2-induced modules that could be therapeutically targeted by repurposing existing and approved drugs. Compared to traditional network-based drug-repositioning methods, our approach offers two key innovations: (1) Understanding the underlying mechanisms linking COVID-19 to CNS disorders could shed light on the observed neurological symptoms and aid in the development of therapeutic strategies. Our method leverages CNS disorder data for comorbidity network construction, providing a perspective on the molecular mechanisms and drug repurposing of COVID-19 from the standpoint of the central nervous system. (2) Computational studies aiming to identify candidate drugs for COVID-19 drug repurposing have used multistage analyses, including network proximity measure analysis, that are focused on disease-related proteins, specifically, and their interactomes. By contrast, our strategy has introduced dynamic information to DTN analysis, allowing for us to consider network dynamics to measure the disturbance of the entire module when a drug acts on its partner proteins. In addition, the use of molecular docking and MD simulations to structurally understand the MOA for the drugs was vital to this investigation.

This study demonstrated that our method is feasible in therapeutic target identification and drug repositioning in COVID-19. By introducing a system-biology-based method, we have elucidated the potential relationship between COVID-19 and four CNS disorders through comorbidity network construction and analysis. Using CMap drug perturbation data to screen therapeutic target modules, particularly those involved in protein synthesis, provides a new avenue for the further treatment of COVID-19. The dynamic-perturbation-network-based drug-repurposing method applied to the drug–target module identified UCHL1 as a potential drug target and suggested pregnenolone and BRD-K87426499 as promising drug candidates for COVID-19. These findings not only offer insights into the neurological implications and the comorbidity conditions of COVID-19 but also enhance drug discovery efforts both during and after the pandemic.

UCHL1 is a neuron-specific protein known for its role in the ubiquitin–proteasome system’s regulation, and its dysregulation has been shown to be associated with CNS disorders. Recent studies have highlighted its potential importance in COVID-19 patients. UCHL1 is an important neurological biomarker in the study of COVID-19, and its level is higher in COVID-19 patients than in non-COVID-19 controls with mild cognitive impairment (MCI) or AD [49]. In severe COVID-19 patients, UCHL1, along with other neurological biomarkers, such as GFAP, NfL, and TAU, can serve as an early predictor of poor prognosis [50]. Another study indicated that autoantibodies, including those against UCHL1, were more prevalent in COVID-19 patients than in controls, particularly among those experiencing altered consciousness [51]. Overall, UCHL1 is closely related to the severity and prognosis of COVID-19 in both the acute and late stages, suggesting its potential as a drug target for COVID-19.

Many candidate drugs have been proposed as repurposable drugs against SARS-CoV-2 infection, such as a group of kinase inhibitors [26,52], estrogen receptor modulators [53], microtubule-regulating agents [54], HCV inhibitors [55], and H1 antihistamines [27]. Notably, several drugs active in the CNS have been identified as repurposable for COVID-19, following SAveRUNNER analysis, such as dopaminergics and dopamine antagonists [56]. Herein, we proposed a series of dopamine-related drugs as potential drugs for COVID-19 treatment, especially pregnenolone and BRD-K87426499. Pregnenolone, as a dopamine release enhancer, is closely linked to the role of UCHL1 in regulating the dopamine system. UCHL1 may affect the function of dopaminergic neurons by participating in the processing of alpha-synuclein and reducing its accumulation, thereby inhibiting the production of alpha-synuclein aggregates in PD. Concurrently, it promotes dopamine release in the SCNS, which can alleviate local and systemic damage to the dopamine system and slow the progression of AD. This drug’s MOA in AD and PD suggests that pregnenolone and BRD-K87426499 may have a similar therapeutic effect in treating COVID-19. Our study included four CNS disorders, which can be divided into three categories based on the age of the onset. The first category includes PD and AD, which primarily occur in the elderly. The second category is MS, mainly affecting individuals aged 20–40. The third category is ASD, which predominantly occurs in children. According to the investigation of the target (UCHL1) and the MOA analysis of the two drugs, we recommend prioritizing research on CNS disorders in elderly patients with long COVID.

This study has limitations both in methodology and application. First, more high-quality comprehensive data are needed for accurate network construction and analysis. COVID-19 has caused a broad spectrum of clinical manifestations, ranging from asymptomatic to severe distress syndromes. In addition, COVID-19 has evolved different mutation lineages. On the other hand, both CNS disorders and COVID-19 are age associated. A pressing challenge is how to integrate diverse clinical phenotype data, mutation data, and epidemiological data to optimize existing comorbidity network models, such as by considering overlaps between CNS disorders and COVID-19 severity. Second, effective drug verification is needed. This study proposed two new drug candidates for the treatment of CNS disorders or COVID-19. One of the recommended drugs is a steroid, pregnenolone, while other steroids, such as the glucocorticoid dexamethasone, are also used. However, because of the pleiotropic effects of dexamethasone, it is only recommended for severe cases and should be avoided in mild cases [57]. Additionally, the inappropriate use of dexamethasone for COVID-19 has been reported to lead to adverse outcomes, particularly among individuals with diabetes, obesity, and insulin resistance [58]. Pregnenolone shares a similar side effect profile with other steroids, making it challenging to determine its specific side effects in the target patient population, such as elderly individuals with CNS disorders. Future research should focus on validating the use of pregnenolone and BRD-K87426499 through experimental studies and clinical trials to confirm the efficacy.

## 4. Materials and Methods

### 4.1. Collection of Disease-Associated Proteins

Prior to the construction of the comorbidity network, we gathered a comprehensive set of genes associated with COVID-19 and central nervous system (CNS) diseases. COVID-19-associated proteins were obtained from three publicly available data resources. Gordon et al. [33] and Zhou et al. [34] constructed two human SARS-CoV-2–protein interaction maps, providing highly reliable interacting proteins. Additional data were taken from a highly reliable differential gene set of SARS-CoV-2, which is closely related to the pathogenesis of the COVID-19 disease and reveals potential targets for drug repurposing [35]. To collect the CNS-disorder-related proteins, a systematic literature review between COVID-19 and CNS disorders was performed by searching the PubMed database (accessed in December 2023). A total of 13 CNS disorders were considered, including Alzheimer’s disease (AD), Parkinson’s disease (PD), multiple sclerosis (MS), autism spectrum disorder (ASD), epilepsy (EP), schizophrenia (SZ), attention deficit hyperactivity disorder (ADHD), bipolar disorder (BD), brain tumors, Huntington’s disease (HD), and vegetative disorder. The number of papers co-reporting each disease with COVID-19 was statistically determined using search combinations, such as “COVID-19” and “the name of CNS”, appearing simultaneously in the title or abstract. Next, focusing on the top-ranked CNS disorders, disease-associated proteins were retrieved from OMIM (https://www.omim.org/), PharmGKB [59] (https://www.pharmgkb.org/), PheGenI (https://www.ncbi.nlm.nih.gov/gap/phegeni), and DisGeNET [60] (https://www.disgenet.com/) databases.

### 4.2. Network Construction and Modular Analysis

Human interactome data were downloaded from the STRING database [61] and filtered for PPIN using a comprehensive score of ≥400. The shared-comorbidity genes were mapped to the PPIN to construct a comorbidity network. Node- and edge-prioritization-based community analysis (ne-PCA) [62] was then utilized to identify functional modules in this network. The ne-PCA integrates the top-down Girvan–Newman (GN) algorithm [63] with the complementary bottom-up label propagation algorithm (LPA) [64], employing the hypergeometric test to assess the correlation between the modules identified using the GN and LPA. The common elements of the modules with significant *p*-values of less than 0.05 across all the modules are determined to be robust modules.

The topological–functional connection (TFC) score [65,66] incorporates known functional information into the edges of the network to measure the role of the edges in signal transduction and is defined as follows:

(1) $$TFC=\sum_{n=1}^{N}\frac{T_{n}^{*}+F_{n}}{\left|T_{n}^{*}+F_{n}-2\right|}*100$$

(2) $$T_{n}^{*}=\frac{T_{n}-{Min}_{T}}{{Max}_{T}-{Min}_{T}}$$

where *N* is the number of interactions, and $T_{n}$ and $F_{n}$ represent the edge betweenness and GO semantic similarity of interaction *n*. We calculated the TFC scores for all the PPIs within the modules, using the average TFC score to gauge the efficiency of the signal transmission within the PPIN of the module.

### 4.3. Functional Enrichment Analysis

GOBP and KEGG pathway enrichment analyses were used for the functional annotation of each network module, by applying the enrichGO and enrichKEGG functions of the R package ClusterProfiler (v4.8.1) [67]. The hypergeometric distribution was used to estimate whether a list of genes was significantly enriched in each GO/KEGG pathway. BH-adjusted *p*-values of <0.05 and q-values of <0.2 were used to identify significantly enriched terms.

### 4.4. Screening of Drug–Response Modules

We employed the CMap database to identify potential drug–response modules. CMap (https://clue.io) utilized the compound perturbation signature from CMap LINCS 2020 [68,69], which consists of a sorted list of genes reflecting gene expression patterns under different conditions, by applying the Kolmogorov–Smirnov (KS) test to calculate the ranking positions of query genes in the drug perturbation signature, resulting in connectivity scores ranging from −1 to +1. The closer the score is to −1, the stronger the compound’s ability to reverse the gene expression. In this way, CMap can identify compounds that can effectively reverse the expressions of disease-related genes. We uploaded all the genes in the module, as upregulated genes, to the CMap server and evaluated the module’s drug response ability by calculating the connectivity scores.

### 4.5. Weighted Drug–Target Network

To construct the drug–target network (DTN), we used level 5 shRNA perturbation expression profile and compound perturbation expression profile data from CMap LINCS 2020. The H1299, A549, Calu-3, NCI-H358, and NCIH2073 cell lines were selected, and the Spearman correlation coefficients were calculated for the correlations between the shRNA perturbation expression profile and all the compound perturbation expression profiles for all the genes with shRNA data in the module. The calculation formula is as follows [70]:

(3) $$\rho =1-\frac{6\sum d_{i}^{2}}{n\left(n^{2}-1\right)}$$

where *n* represents the total number of genes, and $d_{i}$ represents the level difference of the same gene between the shRNA perturbation expression profile and the compound perturbation expression profile; ρ = 1 indicates that two variables have a completely positive correlation, and ρ = 0 indicates that there is no linear correlation between two variables. We selected the top 1% of the compounds targeting each gene in the correlation ranking as potential drugs, which were considered to have interactions with the genes, thereby establishing an initial unweighted DTN. Subsequently, we employed DeepPurpose to assign weights to the DTN, reflecting the affinities between the drugs and their target genes [71]. DeepPurpose takes the SMILES format of the drug and the amino acid sequence of the target as inputs, which come from the PubChem database and Uniprot database, respectively.

### 4.6. Perturbation-Response-Scanning Analysis

Based on the weighted DTN, PRS considers the affinities of drugs to their targets as a form of perturbation and records the displacements of all the targets as the responses [72,73], which enable us to evaluate how the nodes within the module react to external disturbances. Hence, we represented the drug’s affinity for target x as a force vector ($F_{d_{x}}$). The PRS matrix ($S_{PRS}$) in the module was computed using ProDy [74], which element $p_{ij}$ signifies the response of node j to the perturbation at node i. Subsequently, the perturbation effect of the drug binding to its target protein was computed by performing a matrix multiplication of the force vector and the PRS matrix, which is denoted as follows:

(4) $$F_{d_{x}}={\left[0\;0\;0\cdots f_{d_{x}}\cdots 0\;0\;0\right]}^{T}$$

(5) $${∆R}_{DTI}\;=\;S_{PRS}*F_{d_{x}}$$

where $S_{PRS}$ is the identity matrix with a consistent force on each node, and $f_{d_{x}}$ is the specific affinity of the drug for protein *x*. The perturbation score (ps) for the drug is represented as the average perturbation of all the nodes in the network, capturing the effect of the *DTI* on the entire DTN comprehensively.

### 4.7. RNA-Seq Data Analysis

Two independent COVID-19 transcriptome data were used to study the expression of the potential target [43]. The GSE152418 dataset comprises transcriptome profiles from 17 COVID-19 patients and 17 healthy controls, obtained from peripheral blood mononuclear cells. Similarly, the GSE190496 dataset includes transcriptome data from lung tissue, with samples from 6 individuals with normal lung function and 13 COVID-19 patients. Differential expression analysis was performed using the R package Limma (v3.52.4). A linear model was fitted to the data before empirical-Bayes-moderated t-statistics were calculated, and multiple-testing correction (Benjamin–Hochberg) was performed. Genes with log_2_(FC) > 2 and a *p*-value of <0.05 were considered as differentially expressed genes (DEGs).

### 4.8. Drug Annotation

The indication and mechanism-of-action (MOA) information of the drugs was retrieved from Drugbank [75] (https://www.drugbank.ca/) and CMap LINCS 2020, and the clinical research status of the drugs was searched through the ClinicalTrials.gov portal’s website.

### 4.9. Molecular Docking

Molecular docking was performed using Autodock Vina to estimate the combining tendencies between the candidate targets and their correspondingly repositioned drugs [76]. Protein structures downloaded from the AlphaFold database [77] and the 3D structures of small-molecule drugs obtained from the PubChem and ChemSpider [78] databases were utilized for semi-flexible docking. As a result, the complex conformations with the lowest docking energies ($∆G_{Docking}$) were obtained.

### 4.10. Molecular Dynamics Simulations

To further elucidate the interaction mechanisms between potential targets and drugs, 500 ns MD simulations were conducted through GROMICS [79], under the conditions of the Amber force field with GAFF parameters [80]. Specifically, each system was immersed in a dodecahedral solvent box filled with TIP3P [81] water molecules and then automatically neutralized using NA^+^ or CL^−^, and energy minimization and equilibration were performed using default settings subsequently. Once the system reaches equilibrium, the binding free-energy ($∆G_{MMGBSA}$) is calculated using the MMGBSA [82] approach to assess the stability of the drug–protein complexes and is defined as follows:

(6) $${∆G}_{MMGBSA}={∆E}_{MM}+{∆G}_{sol}-T∆S$$

where ${∆E}_{MM}$, ${∆G}_{sol}$, and T∆S represent the changes in the gas-phase MM energy, solvent-free energy, and conformational entropy upon binding, respectively. Next, RMSD/RMSF and hydrogen bond changes were calculated using gmx programs and Visual Molecular Dynamics [83], respectively, providing detailed insights into the dynamics of the complex. Additionally, we performed MD simulations on the apo system of the candidate target and calculated the RMSD and RMDF as a control.

## 5. Conclusions

Our integrated study highlights shared mechanisms between COVID-19 and CNS disorders and opens promising avenues for the drug repurposing of long COVID. By employing data-driven target module identification combined with network dynamic perturbation analysis, we identified a potential target and two promising drug candidates for COVID-19. The findings suggest that our system-biology-based workflow offers a robust framework for drug discovery in complex diseases.

## Figures and Tables

**Table 1:** List of drugs targeting UCHL1.

Drug	Δ*G_D__ocking_* (kcal/mol)	Indication	MOA	Clinical Trial
pregnenolone	−7.7	NA	dopamine release enhancer	Phase 2—ASD
methysergide	−7.2	vascular headache	serotonin receptor antagonist	
flutamide	−6.5	prostate cancer	androgen receptor antagonist	
propantheline	−6	antimuscarinic agent	acetylcholine receptor antagonist	
zileuton	−5.9	chronic asthma	lipoxygenase inhibitor	Phase1—Pneumonia
benactyzine	−5.7	depression and related anxiety	acetylcholine receptor antagonist	
lidocaine	−5.4	amide-based anesthetic	histamine receptor agonist	Phase3—COVID-19
pseudopelletierine	−4.9	NA	anthelmintic	
norepinephrine	−4.8	vasovagal shock	adrenergic receptor agonist	
BRD-K95482240	−9.3	NA	tubulin polymerization inhibitor	
BRD-K62493605	−8.4	NA	dopamine receptor antagonist	
BRD-K35708212	−8.4	NA	BCL inhibitor	
BRD-K87426499	−8.1	NA	dopamine receptor antagonist	
BRD-A26595446	−8	NA	cyclooxygenase inhibitor	
BRD-K46267071	−8	NA	NA	
BRD-K83151319	−7.8	NA	PI3K inhibitor	
BRD-K33586157	−7.6	NA	cyclooxygenase inhibitor	
BRD-K82857306	−7.5	NA	MEK inhibitor	
BRD-K18587499	−7.3	NA	histamine receptor antagonist	
BRD-K48924007	−6.8	NA	dopamine receptor antagonist	
BRD-K00859168	−6.5	NA	topoisomerase inhibitor	
BRD-K99311057	−6.2	NA	cyclooxygenase inhibitor	
BRD-K04188046	−5.8	NA	adrenergic receptor antagonist	
BRD-A96272097	−5.2	NA	dopamine receptor antagonist	

Indications and MOA information are obtained from Drugbank and LINCS databases, and the clinical research status of the drugs is obtained from the ClinicalTrials portal (https://www.clinicaltrials.gov/).

## Data Availability

The original contributions presented in this study are included in the article/Supplementary Materials; further inquiries can be directed to the corresponding authors.

## Supplementary Materials

The supporting information can be downloaded at https://www.mdpi.com/article/10.3390/ijms25168917/s1.
