# Supplementary material for: Drug Repurposing for COVID-19 by Constructing a Comorbidity Network with Central Nervous System Disorders
Source: Int J Mol Sci. 2024 Aug 16;25(16):8917. doi: 10.3390/ijms25168917 (PMC11354300; doi:10.3390/ijms25168917)
Supplement: Supplementary file 1 [file ijms-25-08917-s001.zip › ijms-3136905-supplementary.pdf]

International Journal of
Molecular Sciences

MDPI

Article

Drug Repurposing for COVID-19 by Constructing a Comorbidity Network with Central Nervous System Disorders

Jing Qian [1,†], Bin Yang [1,†], Shuo Wang [1], Su Yuan [1], Wenjing Zhu [1], Ziyun Zhou [1], Yujuan Zhang [2,*] and Guang Hu [1,3,4,5,*]

1 MOE Key Laboratory of Geriatric Diseases and Immunology, Suzhou Key Laboratory of Pathogen Bioscience and Anti-Infective Medicine, Department of Bioinformatics, Center for Systems Biology, School of Life Sciences, Suzhou Medical College of Soochow University, Suzhou 215213, China; qianjing03223@163.com (J.Q.); biny12212021@163.com (B.Y.); 2130403023@stu.suda.edu.cn (S.W.); 2230401047@stu.suda.edu.cn (S.Y.); 2230805006@stu.suda.edu.cn (W.Z.); zhouziyun1900@hotmail.com (Z.Z.)
2 Experimental Center of Suzhou Medical College of Soochow University, Suzhou 215123, China
3 Jiangsu Province Engineering Research Center of Precision Diagnostics and Therapeutics Development, Soochow University, Suzhou 215123, China
4 Key Laboratory of Alkene-Carbon Fibres-Based Technology & Application for Detection of Major Infectious Diseases, Soochow University, Suzhou 215123, China
5 Jiangsu Key Laboratory of Infection and Immunity, Soochow University, Suzhou 215123, China
* Correspondence: zhangyujuan@suda.edu.cn (Y.Z.); huguang@suda.edu.cn (G.H.)
† These authors contributed equally to this work.

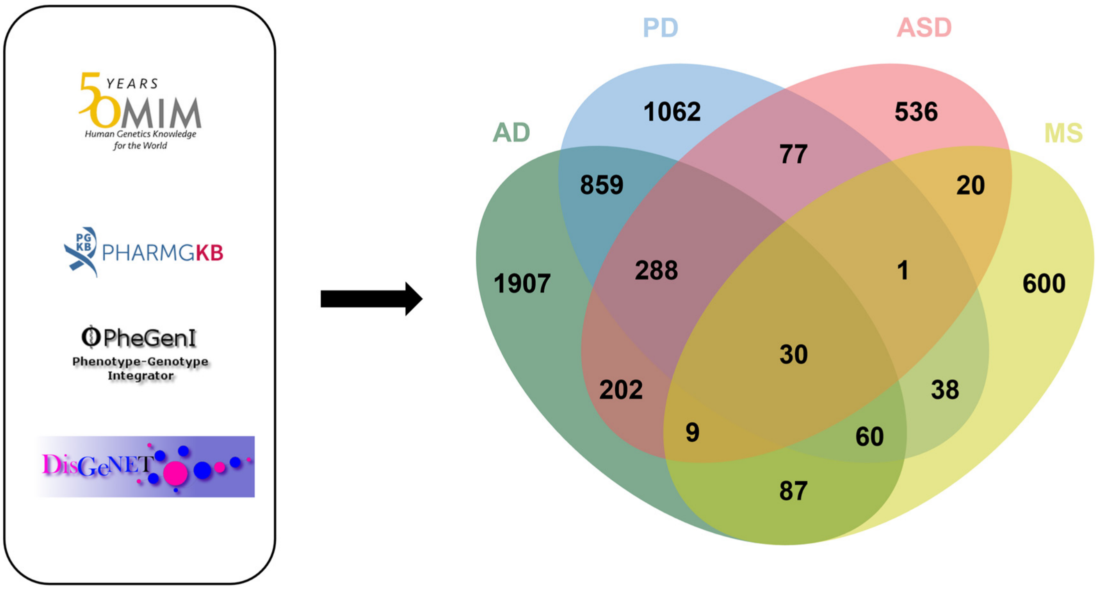

Figure S1. Data sources and the numbers of COVID-19 related proteins and genes related to the four CNS-disorders.

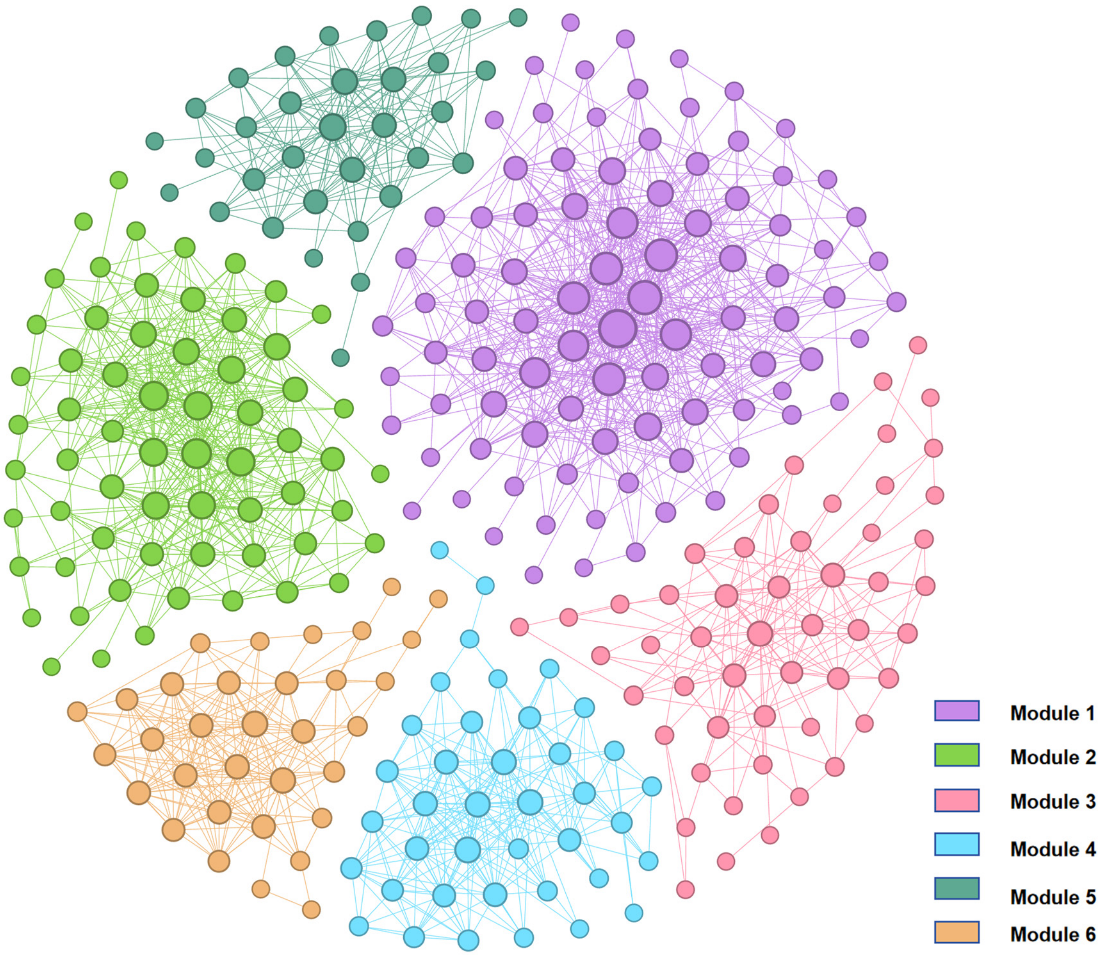

Figure S2. Six major modules of the comorbidity network. Purple, light green, pink, blue, dark green, and orange represent Modules 1, 2, 3, 4, 5, and 6, respectively. The size of each node corresponds to its degree, with larger nodes indicating higher degrees.

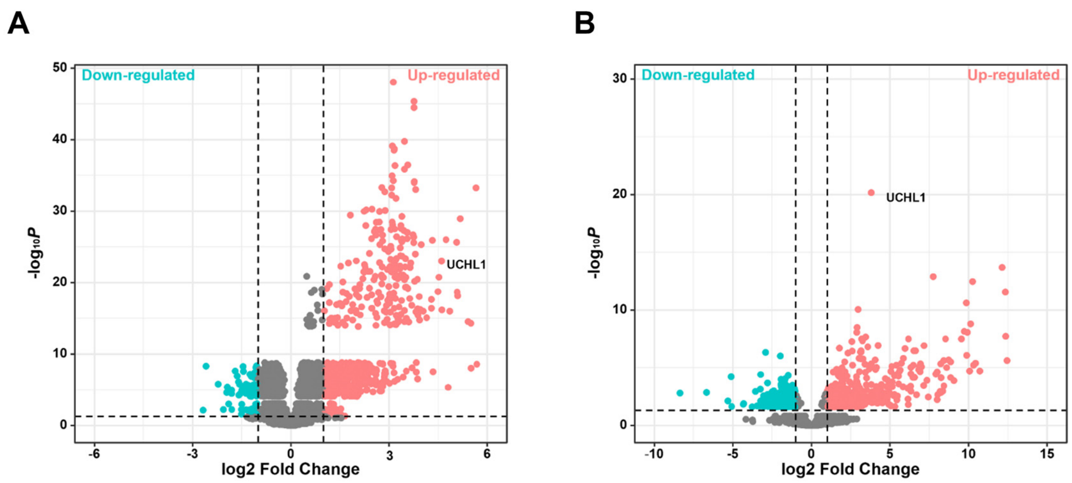

Figure S3. Volcano plots illustrating the variance in gene expression between COVID-19 patients and controls are shown for the GSE152418 and GSE190496 datasets. (A) The GSE152418 dataset includes transcriptome profiles from 17 COVID-19 patients (12 severe cases, 4 mild cases, and 1 convalescent case) and 17 healthy controls, all obtained from peripheral blood mononuclear cells. (B) The GSE190496 dataset comprises transcriptome data from lung tissue, with samples from 6 individuals with normal lung function and 13 COVID-19 patients (11 postmortem cases and 2 survivors). In both datasets, UCHL1 demonstrates a significant upregulation in the COVID-19 patient group.

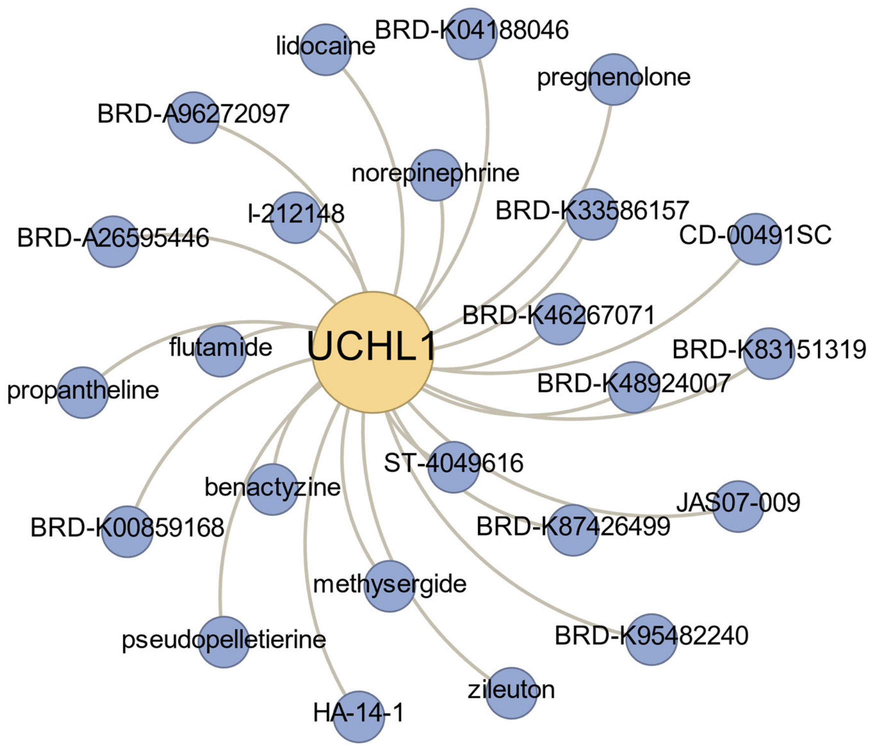

Figure S4. The UCHL1-drug network.

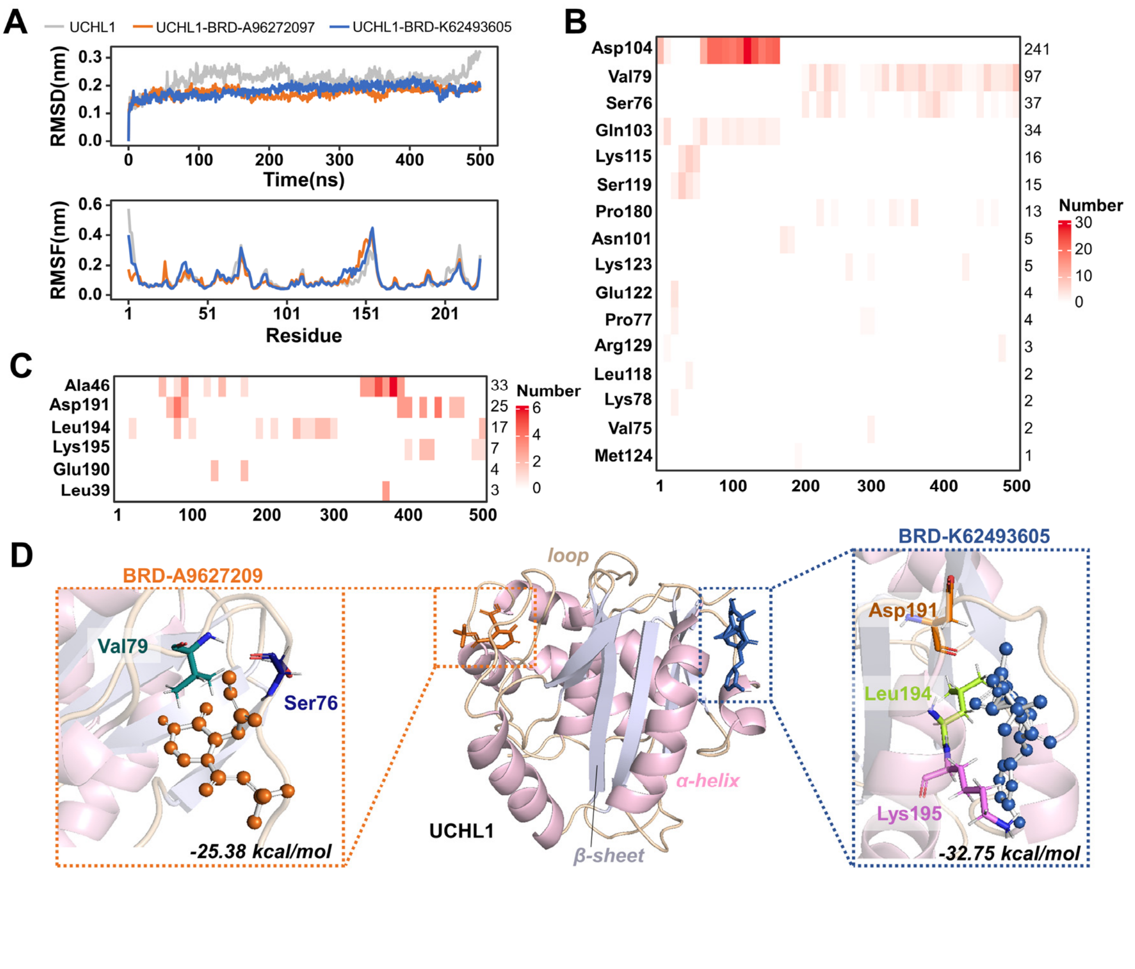

Figure S5. 500 *ns* MD simulations of UCHL1-compound complexes. (A) RMSDs and RMSFs of the UCHL1 apo system and the systems of UCHL1 complexed with BRD-A96272097 and BRD-K62493605, respectively. (B) and (C) depict the hydrogen bonding connection between BRD-A96272097 and BRD-K62493605 to UCHL1. Changes in color intensity indicate the frequency and intensity of these interactions. (D) The specific conformation of ligand molecules in protein binding pockets. Important interacting residues and binding free energy after stabilization are marked.

Table S4. The binding free energies between four drugs and UCHL1 by MMGBSA.

Energy Component	pregnenolone	BRD-K62493605	BRD-K87426499	BRD-A96272097
ΔBOND	0	0	0	0
ΔANGLE	0	0	0	0
ΔDIHED	0	0	0	0
ΔVDWAALS	-37.44	-37.59	-54.12	-28.56
ΔEEL	0	0	0	0
Δ1-4 VDW	0	0	0	0
Δ1-4 EEL	0	0	0	0
ΔEGB	8.17	7.84	12.82	6.29
ΔESURF	-3.91	-3.01	-5.11	-3.11
ΔGGAS	-37.44	-37.59	-54.12	-28.56
ΔGSOLV	4.25	4.84	7.71	3.18
ΔTOTAL	-33.19	-32.75	-46.41	-25.38
